# Supplementary material for: IDO1-Targeted Therapy Does Not Control Disease Development in the Eµ-TCL1 Mouse Model of Chronic Lymphocytic Leukemia
Source: Cancers (Basel). 2021 Apr 15;13(8):1899. doi: 10.3390/cancers13081899 (PMC8071295; doi:10.3390/cancers13081899)
Supplement: Supplementary file 1 [file cancers-13-01899-s001.zip › cancers-1149775-supplementary.pdf]

cancers

MDPI

Supplementary Materials: IDO1-Targeted Therapy Does Not Control Disease Development in the Eμ-TCL1 Mouse Model of Chronic Lymphocytic Leukemia

Selcen Öztürk, Verena Kalter, Philipp M. Roessner, Murat Sunbul and Martina Seiffert

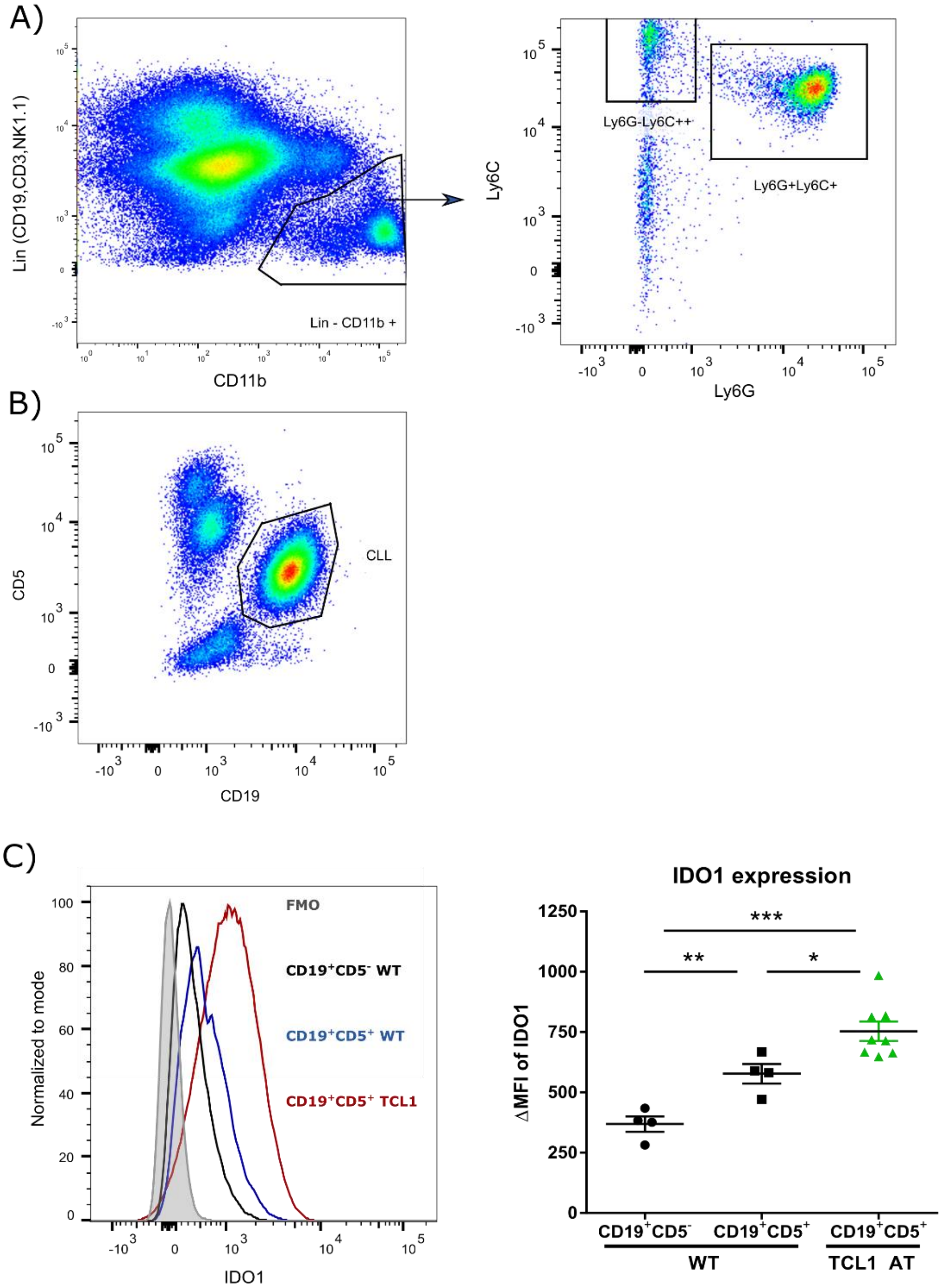

Figure S1. Gating strategies related to Fig. 1 and IDO1 expression in B cell subsets. A) Gating strategy for flow cytometry analysis of myeloid cells depicted in Figure 1A-D. **B)** Gating strategy for CLL cells. **C)** Representative histogram and quantification of IDO1 expression in CD5+CD19+ and CD5-CD19+ B cells from tumor-free wild-type (WT) mice, as well as CD5+CD19+ tumor cells in mice after adoptive transfer of TCL1 leukemia cells (TCL1 AT). All graphs show means ± SEM. Comparison of groups was performed with Welch's t-test. *p<0.05, **p<0.01, ***p<0.001. MFI = normalized median fluorescence intensity.

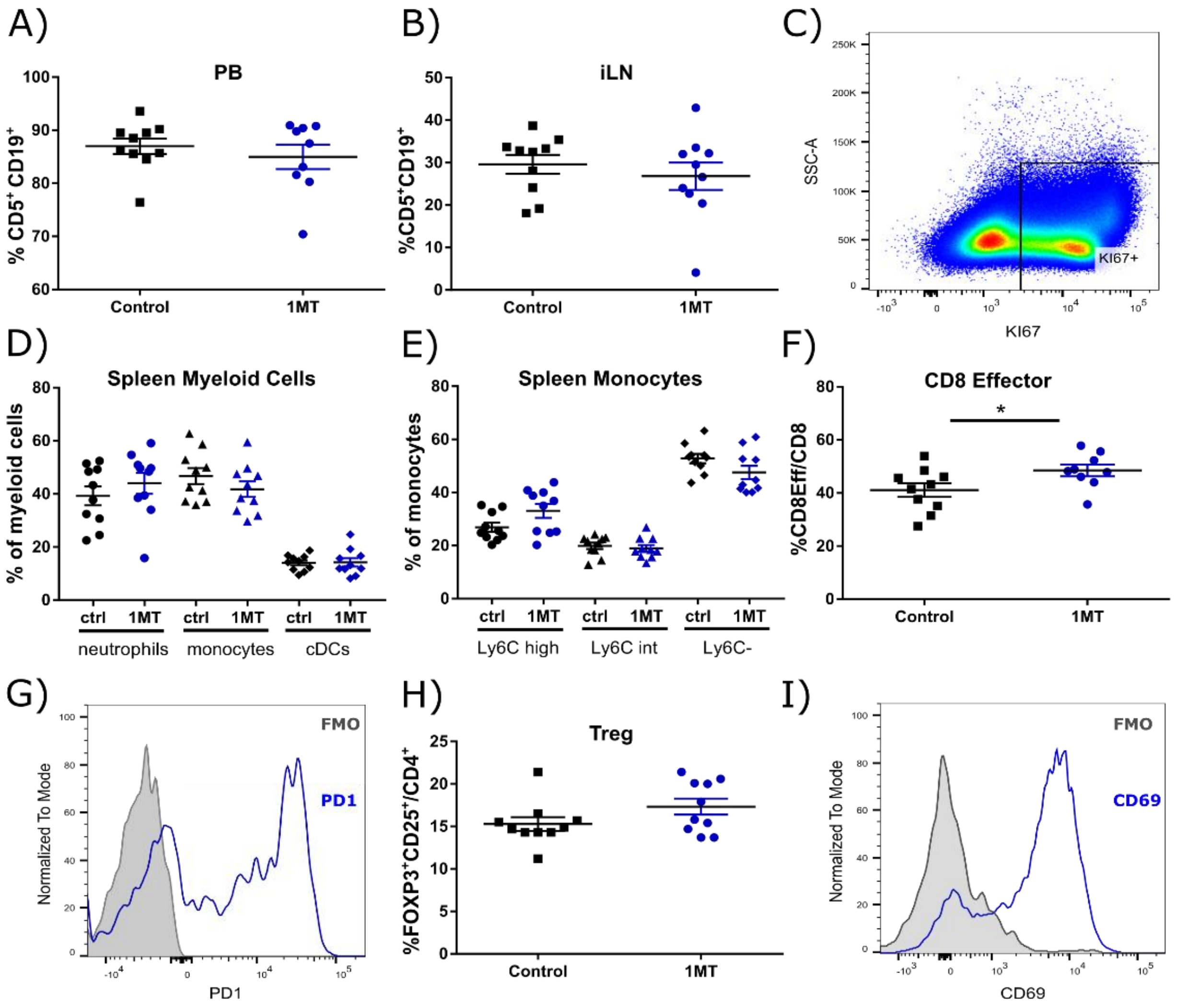

Figure S2. 1MT treatment has minor effects on CLL development and tumor-associated immune cells. Wild-type mice were transplanted i.p. with 2×10^7 TCL1 tumor cells and were divided into 2 groups (n=10) directly after transplantation. Mice of the 1MT group had continuous access to the drug via drinking water. Mice of the control group (Con) received vehicle in the drinking water. Flow cytometry analysis was performed 6 weeks after tumor transplantation. **A-B)** Percentages of $CD5^+CD19^+$ CLL cells in peripheral blood (PB) and inguinal lymph nodes (iLN) at the endpoint. **C)** Representative dot plot showing the gating strategy for $KI67^+$ cells. **D)** After exclusion of cells expressing CD19, CD3 or NK1.1, neutrophils were defined as $CD11b^+Ly6G^+$, conventional dendritic cells (cDCs) as $CD11c^{high}MHC\ II^+$, and monocytes as $CD11b^+F4/80^+CD11c^{-/low}$ cells. Frequencies of these populations in the spleen out of total myeloid cells are depicted. **E)** Splenic monocyte subsets were further analyzed by their Ly6C expression and frequencies of cells with high, intermediate (int) and no Ly6C expression are depicted. **F)** $CD8^+$ effector T cells in the spleen were quantified as CD127lowCD44+ cells. **G**) Representative histogram showing PD-1 staining on splenic CD8+ effector T cells. **H**) Quantification of CD25+FOXP3+ Tregs in the spleen. **I**) Representative histogram showing CD69 staining on splenic Tregs. All graphs show means ± SEM. Comparison of groups was performed with Welch's t-test. Statistics were only indicated when significant. *p<0.05. FMO = fluorescence-minus-one control.

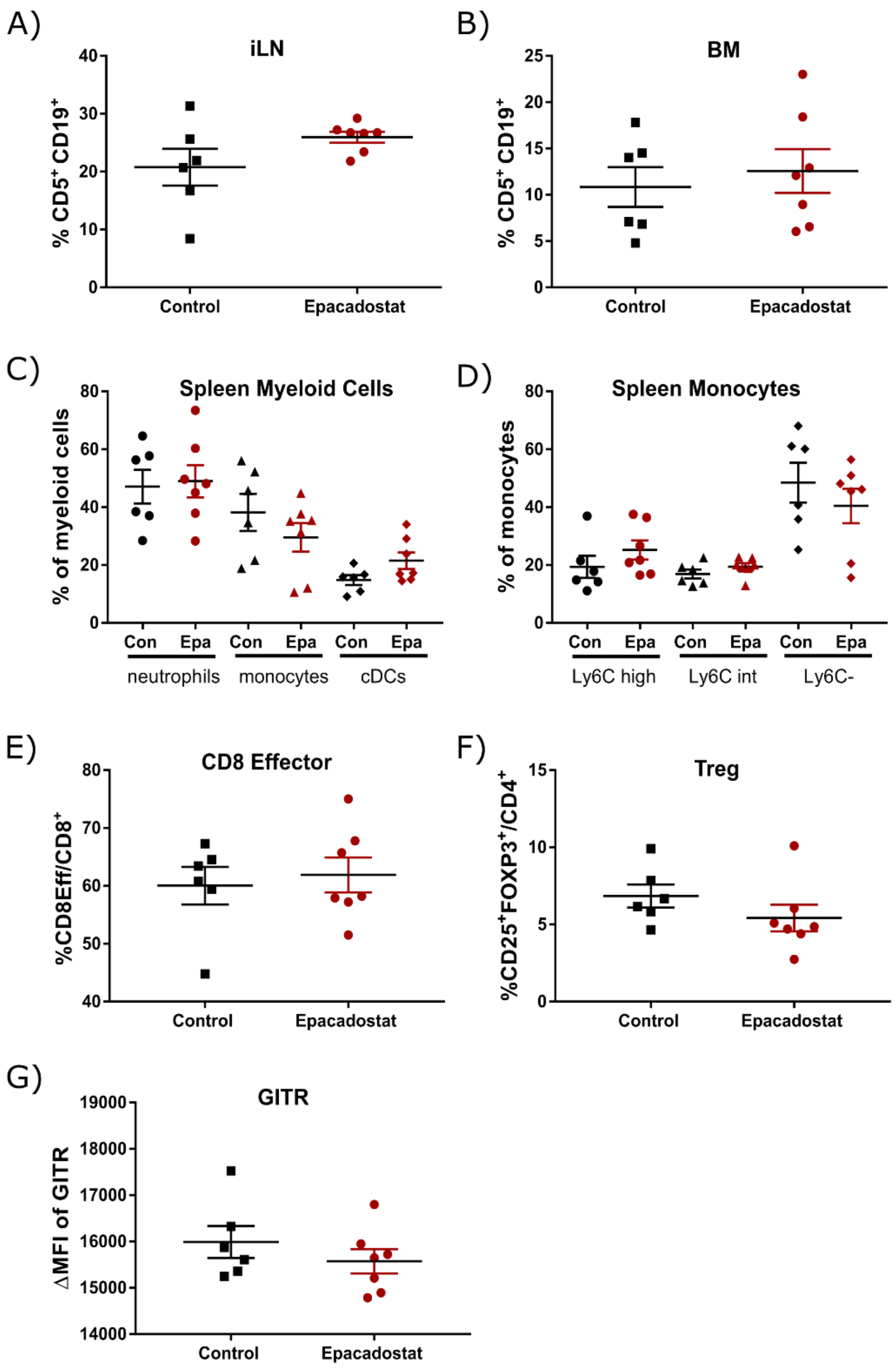

Figure S3. Epacadostat treatment has minor effects on CLL development and tumor-associated immune cells. Wild-type mice were transplanted i.p. with 2×10^7 TCL1 tumor cells and were divided into 2 groups 2 weeks post tumor transplantation. Mice were treated with epacadostat (Epa) or vehicle (Control) by oral gavage once daily and flow cytometry analysis was performed 5 weeks after tumor transplantation (Control, n=6; Epacadostat, n=7). **A-B)** Percentages of $CD5^+CD19^+$ CLL cells in inguinal lymph nodes (iLN) and bone marrow (BM) at the endpoint. **C)** After exclusion of cells expressing CD19, CD3 or NK1.1, neutrophils were defined as $CD11b^+Ly6G^+$, conventional dendritic cells (cDCs) as $CD11c^{high}MHC\ II^+$, and monocytes as $CD11b^+F4/80^+CD11c^{-/low}$ cells. Frequencies of these populations in the spleen out of total myeloid cells are depicted. **D)** Monocyte subsets were further analyzed by their Ly6C expression and frequencies of cells with high, intermediate (int) and no Ly6C expression are depicted. **E)** $CD8^+$ effector T cells in the spleen were quantified as $CD127^{low}CD44^+$ cells. **F)** Quantification of $CD25^+FOXP3^+$ Tregs in the spleen. **G)** Quantification of GITR expression on splenic Tregs. All graphs show means ± SEM. Comparison of groups was performed with Welch's t-test. Statistics were only indicated when significant. MFI = normalized median fluorescence intensity.

Table S1. Antibodies used in this study.

ANTIBODY	SOURCE	IDENTIFIER
Anti-mouse CD11b (clone M1/70)	ThermoFisher	Cat# 25-0112-81, RRID:AB_469587
Anti-mouse CD11c (clone N418)	Biolegend	Cat# 117334, RRID: AB_2562415
Anti-mouse CD127 (clone A7R34)	Biolegend	Cat# 135014, RRID:AB_1937265
Anti-mouse CD19 (clone eBio1D3)	ThermoFisher	Cat# 11-0193-82, RRID:AB_657666, Cat# 12-0193-81, RRID:AB_657661, Cat# 48-0193-82,RRID:AB_2734905
Anti-mouse CD25 (clone PC61)	Biolegend	Cat# 102036, RRID:AB_2563059
Anti-mouse CD3e (clone 145-2C11)	ThermoFisher	Cat# 11-0031-82, RRID:AB_464882
Anti-mouse CD3e (clone 500A2)	BD	Cat# 560801, RRID:AB_2034005
Anti-mouse CD4 (clone GK1.5)	Biolegend	Cat# 100414, RRID:AB_312699
Anti-mouse CD44 (clone IM7)	ThermoFisher	Cat# 56-0441-82, RRID:AB_494011
Anti-mouse CD5 (clone 53-7.3)	ThermoFisher	Cat# 17-0051-81, RRID:AB_469330
Anti-mouse CD5 (clone 53-7.3)	BD	Cat# 563194, RRID:AB_2738061
Anti-mouse CD69 (clone H1.2F3)	Biolegend	Cat# 104508, RRID:AB_313111
Anti-mouse CD8a (clone 53-6.7)	Biolegend	Cat# 100744, RRID:AB_2562609
Anti-mouse F4/80 (clone BM8)	Biolegend	Cat# 123110, RRID:AB_893486
Anti-mouse Foxp3 (clone FJK-16s)	ThermoFisher	Cat# 56-5773-82, RRID:AB_1210557
Anti-mouse GITR (clone DTA-1)	Biolegend	Cat# 126316, RRID:AB_2563384
Anti-mouse IDO1 (clone mIDO-48)	ThermoFisher	Cat# 50-9473-82, RRID:AB_ 2574335
Anti-mouse Ki67 (clone 16A8)	Biolegend	Cat# 652404, RRID:AB_ 2561525
Anti-mouse KLRG1 (clone 2F1/KLRG1)	Biolegend	Cat# 138424, RRID:AB_2564051
Anti-mouse Ly6G (clone 1A8)	ThermoFisher	Cat# 48-9668-82, RRID:AB_2637124
Anti-mouse Ly6C (clone HK1.4)	Biolegend	Cat# 128026, RRID:AB_10640120
Anti-mouse MHC-II I-A/I-E (clone M5/114.15.2)	ThermoFisher	Cat# 56-5321-82, RRID:AB_494009
Anti-mouse NK1.1 (clone PK136)	Biolegend	Cat# 108706, RRID:AB_313393
Anti-mouse PD-1 (clone RMP1-30)	Biolegend	Cat# 109116, RRID:AB_2566548
